# Activation of FADD-Dependent Neuronal Death Pathways as a Predictor of Pathogenicity for LRRK2 Mutations

**DOI:** 10.1371/journal.pone.0166053

**Published:** 2016-11-10

**Authors:** Katerina Melachroinou, Emmanouela Leandrou, Polytimi-Eleni Valkimadi, Anna Memou, Georgios Hadjigeorgiou, Leonidas Stefanis, Hardy J. Rideout

**Affiliations:** 1 Division of Basic Neurosciences, Biomedical Research Foundation of the Academy of Athens, Athens, Greece; 2 Department of Neurogenetics, Institute of Biomedical Research & Technology (CERETETH), Larissa, Greece; 3 Department of Neurology, University of Thessaly School of Medicine, Larissa, Greece; 4 Second Department of Neurology, University of Athens Medical School, Athens, Greece; Universita degli Studi di Padova, ITALY

## Abstract

**Background:**

Despite the plethora of sequence variants in LRRK2, only a few clearly segregate with PD. Even within this group of pathogenic mutations, the phenotypic profile can differ widely.

**Objective:**

We examined multiple properties of LRRK2 behavior in cellular models over-expressing three sequence variants described in Greek PD patients in comparison to several known pathogenic and non-pathogenic LRRK2 mutations, to determine if specific phenotypes associated with pathogenic LRRK2 can be observed in other less-common sequence variants for which pathogenicity is unclear based on clinical and/or genetic data alone.

**Methods:**

The oligomerization, activity, phosphorylation, and interaction with FADD was assessed in HEK293T cells over-expressing LRRK2; while the induction of neuronal death was determined by quantifying apoptotic nuclei in primary neurons transiently expressing LRRK2.

**Results:**

One LRRK2 variant, A211V, exhibited a modest increase in kinase activity, whereas only the pathogenic mutants G2019S and I2020T displayed significantly altered auto-phosphorylation. We observed an induction of detergent-insoluble high molecular weight structures upon expression of pathogenic LRRK2 mutants, but not the other LRRK2 variants. In contrast, each of the variants tested induced apoptotic death of cultured neurons similar to pathogenic LRRK2 in a FADD-dependent manner.

**Conclusions:**

Overall, despite differences in some properties of LRRK2 function such as kinase activity and its oligomerization, each of the LRRK2 variants examined induced neuronal death to a similar extent. Furthermore, our findings further strengthen the notion of a convergence on the extrinsic cell death pathway common to mutations in LRRK2 that are capable of inducing neuronal death.

## Introduction

Missense mutations in the gene encoding leucine-rich repeat kinase 2 (LRRK2) are the most common genetic cause of familial forms of Parkinson’s disease (PD), while non-coding sequence variations in the LRRK2 locus have been linked in genome-wide association studies to the more common sporadic form of the disease [1,2]. From a clinical point of view, PD caused by mutations in LRRK2 is remarkably similar to sporadic forms of the disease [3]. LRRK2 is a large 2527-amino acid protein comprised of multiple enzymatic as well as protein interaction domains, whose function has been implicated in a variety of cellular functions (see [4] for recent review). While many sequence variants have been identified, only relatively few display a definitive causative link to PD (see [5,6] for review), and these mutations are found primarily within the ROCO signaling core of the protein. Several other sequence variants have been reported that alter the risk of developing PD; both increasing the risk as in the case of the G2385R variant [7], or lowering the risk as in the N551K/R1398H/K1423K haplotype [8]. For the remaining sequence variants, particularly where only a few cases are known and the clinical and genetic data concerning pathogenicity are unclear; it can be difficult to definitively conclude whether a given mutation is in fact causative for PD. When examining the biological effects of LRRK2 sequence variants in experimental models, what should be the criteria for considering a particular mutation as likely pathogenic? Moreover, can clear phenotypic profiles evident in experimental models be used to help establish pathogenicity when genetic evidence is lacking or inconclusive?

A number of cellular phenotypes have been described in cell lines or primary neurons over-expressing pathogenic mutant LRRK2; including alterations in kinase or GTPase activity, altered phosphorylation state, changes in cellular localization or oligomerization, alterations in autophagic/lysosomal protein degradation, neurite retraction or suppressed neurite outgrowth, disruption of synaptic vesicle neurotransmission, enhanced phosphorylation of some Rab GTPases [9], and activation of cell death signaling pathways [4]. Among these, however, the only uniform phenotype reported by multiple groups and in multiple assays, is the induction of neuronal death. We asked whether the induction of FADD-dependent neuronal death in a cellular model could be a useful tool in determining pathogenicity in less common sequence variants for which the genetic/epidemiological evidence is unclear. We chose to study three sequence variants identified in several families of Greek origin, in comparison to known pathogenic and non-pathogenic LRRK2 mutations. We find that while each of the rare variants examined were able to induce the apoptotic death of primary cultured neurons to a similar extent as for pathogenic mutant LRRK2; differences were observed in the behavior of these mutant forms in other assays. A common element, however, was that the neuronal death induced by each of the variants exhibited a reliance on the death adaptor protein FADD, as has previously been reported for several pathogenic mutations in LRRK2, suggesting that this is a common feature of mutant forms of LRRK2 capable of inducing neuronal death.

## Materials and Methods

### Plasmids

Wild type and mutant human LRRK2 in N-terminal GFP fusion vectors were used as described [10]. Wild type (WT) LRRK2 cDNA was sub-cloned into the pcDNA3.1(+) plasmid with an N-terminal Flag epitope tag. For introduction of pathogenic PD-linked mutations, disease-associated variants, or functional mutations, the Quikchange II site-directed mutagenesis kit (Agilent Technologies; CA, USA) was used according to the manufacturers instructions. Full length V5-tagged FADD, and leucine-zipper FADD-death domain (lz-DD), were used as previously described [10].

### Cell Lines and Primary Neuronal Cultures

HEK293T cells (ATCC; Wesel, DE) were cultured in DMEM (Sigma; MO, USA) medium supplemented with 10% FBS and antibiotics, and transiently transfected in 10cm or 6-well tissue culture plates using calcium phosphate:DNA precipitates. In co-expression experiments, Flag-LRRK2 and V5-FADD were transfected at a ratio of 4:1. Primary embryonic rat cortical neurons were prepared as described [10]. Dissociated neurons were grown at a density of approximately 100,000 neurons/cm^2^ on poly-D-lysine coated glass coverslips in Neurobasal medium containing B-27 serum free supplements (Invitrogen; NY, USA) and penicillin/streptomycin. The use of animals in this study was reviewed and approved by the Institutional Animal Care and Use Committee of BRFAA. Following four days *in vitro*, neurons were co-transfected with Flag-LRRK2 constructs and pcms-EGFP at a ratio of 4:1 using Lipofectamine 2000 (Invitrogen) according to the manufacturer’s instructions. Survival of primary cortical neurons expressing LRRK2 was determined as follows: fixed neurons were immunostained for GFP together with active caspase-3 and Hoechst/DAPI to label apoptotic neurons. Parallel coverslips were double immunostained for GFP (chicken polyclonal; Abcam; Cambridge, UK) and Flag (mouse monoclonal, clone M2; Sigma) or LRRK2 (rabbit monoclonal, clone c41-2; Epitomics/Abcam) to confirm that the majority of GFP-positive cells also over-expressed Flag-LRRK2. Under these conditions, greater than 85% of GFP-positive neurons were also positive for Flag. For quantification, apoptotic neurons were defined as those having condensed fragmented chromatin comprised of two or more apoptotic bodies. More than 100 neurons per coverslip were assessed in triplicate coverslips in a blinded fashion, from three to four independent cultures. The data are presented as the percentage of GFP-positive neurons containing apoptotic nuclear features.

### Antibodies

Chicken anti-GFP (Abcam) was used to detect pcms-EGFP co-transfected in primary neurons. Mouse (clone M2) and rabbit anti-Flag and anti-V5 antibodies were from Sigma. Rabbit anti-phospho-ERM (Cell Signaling Technology; MA, USA) was used in an ELISA to detect phosphorylated LRRKtide. Rabbit monoclonal anti-LRRK2 (clone #c41-2; and clone UDD3; Epitomics/Abcam) was used to label LRRK2 by Western immunoblot. For the detection of phosphorylated LRRK2 we employed: rabbit monoclonal anti-pS935, (from Epitomics/Abcam), and rabbit polyclonal anti-pS1292 (generously provided by Genentech; [11]). For the detection of activated caspase-3, fixed cells were immunostained with active caspase-3 (R&D Systems; MN, USA).

### Size-Exclusion Chromatography and Western Immunoblotting

For the separation of globular protein complexes by SEC, HEK293T cells transiently expressing GFP- or Flag-tagged LRRK2 were washed and collected in ice-cold PBS, centrifuged and re-suspended in PBS containing protein and phosphatase inhibitors and incubated on ice for 20 min. The cells were then disrupted by homogenization in a glass Dounce homogenizer and centrifuged again at low speed (2000 rpm for 10 min) to remove nuclei and un-broken cells. The clarified lysate (3–5 mg total protein) was then injected into a 500 μl loop and separated using PBS as the running buffer through a Superose 6 10/300 column. Fractions (250 μl) were collected in a 96 well plate and stored at -80°C until use. In parallel, native protein molecular weight markers (thyroglobulin, 669kDa & apoferritin, 443kDa are shown in relation to the fractions probed for LRRK2) were run under identical conditions and their elution used as a reference for the approximate size of LRRK2-containing protein complexes. To detect the elution of LRRK2 in each fraction, 45 μl from each fraction was assessed by dot-blot using anti-Flag or anti-GFP antibodies. For quantification, triplicate blots were scanned and the band intensity measured by ImageJ.

Alternatively, the elution of Flag-tagged LRRK2 in SEC fractions was quantified by sandwich ELISA using Flag-coated ELISA plates (see also below for kinase activity assay), coupled with detection using rabbit monoclonal anti-LRRK2 (UDD3). Cell extracts prepared as above from HEK293T cells over-expressing WT or mutant Flag-LRRK2 were fractionated identically as described above by SEC. A 5 μl aliquot of each fraction was bound to the Flag-coated wells, and following extensive washing, detected with rabbit anti-LRRK2 and HRP-conjugated anti-rabbit antibodies. In parallel, a standard curve was established using known amounts of full-length Flag-tagged WT LRRK2 (ThermoScientific), in order to express the LRRK2 present within each fraction in ng/ μl.

In some experiments, soluble cytoplasmic protein was extracted by incubation in a buffer (85 mM PIPES, 10mM EDTA, 1mM MgCl_2_) containing 0.1% Triton for up to 10 min, followed by gently washing in PBS and fixation in 4% paraformaldehyde and immunostaining as described. Confocal images were obtained on a Lecia SP5 microscope as optical slices and then re-constructed into a complete image stack using the image acquisition software.

### Measurement of GTP binding

For determination of GTP binding of the different LRRK2 constructs, 1 mg of clarified total protein lysate of HEK293T cells transiently over-expressing LRRK2 was diluted in IP lysis buffer and incubated with GTP-bound agarose (Sigma) overnight at 4°C under gentle rotation. The beads were centrifuged at 13000 rpm for 2 min, and washed 4X with IP lysis buffer, followed by elution with 2X SDS sample buffer containing 5% beta-mercaptoethanol at 95°C for 5 min. The beads were centrifuged and the supernatant collected and separated by SDS-PAGE. The blotted membranes were probed with anti-Flag or anti-LRRK2 antibodies as indicated.

### Measurement of LRRK2 kinase activity

For assessment of LRRK2 kinase activity, we performed a modification of the assay described by Kamikawaji and colleagues [12]. Briefly, HEK293T cells grown in 6-well plates were transfected with Flag-tagged LRRK2 constructs (as indicated). Seventy-two h following expression, cells were collected in ice-cold PBS, re-suspended in IP lysis buffer, incubated on ice for 20 min, and then centrifuged at 13000 rpm for 10 min. The supernatant was collected and clarified lysate added to Flag-coated 96-well plates (Genscript; NJ, USA) and incubated for 2 h at 37°C while shaking. The wells were washed 3X with ELISA wash buffer (50 mM Tris, pH 7–4; 150 mM NaCl; 0.1% Tween-20), followed by 2 washes with kinase reaction buffer (20 mM Tris, pH 7.5; 20 mM NaCl; 10 mM MgCl_2_; 2 mM DTT). For the kinase reaction, reaction buffer containing 100 μM ATP, 5 μM biotinylated LRRKtide (Innovagen; Lund, SE), and protease/phosphatase inhibitors (Roche) was added to each well and incubated for 30 min at 30°C. The reaction was stopped by the addition of 450 μl of ice-cold binding buffer (50 mM Tris, pH 7.6; 150 mM NaCl; 0.5% NP-40; 20 mM EDTA). Fifty μl of the diluted reaction was added to streptavidin-coated 96-well plates (Thermo Scientific; PA, USA). The plates were incubated at 37°C for 1 h, followed by 4 washes with ELISA wash buffer. The wells were then incubated with anti-phospho-LRRKtide (Cell Signaling) for 1 h at room temperature, followed by 4 washes, and incubation with HRP-conjugated anti-rabbit secondary antibodies for 1 h at room temperature. The wells were washed and incubated for 5 min with chemiluminescent substrate (Thermo Scientific). As a control, cells overexpressing D1994A “kinase-dead” LRRK2 were similarly assessed. For a more precise normalization of kinase activity to LRRK2 expression, the levels of LRRK2 were quantified directly in the 96-well plate by ELISA. Following the kinase reaction, the reaction mix containing phosphorylated LRRKtide was removed, and the wells washed 4X with ELISA wash buffer. The same wells were then incubated with rabbit anti-LRRK2 (Abcam; clone UDD3) for 1 hr at room temperature, followed by 4 washes, and incubation with HRP-conjugated anti-rabbit secondary antibodies (Agilent Technologies/DAKO; CA, USA) for 1 h at room temperature. The wells were washed again, and incubated for 5 min with chemiluminescent substrate (Thermo Scientific). Thus, LRRK2 kinase activity, as determined by the relative amount of phosphorylated LRRKtide, is expressed as a ratio to the amount of LRRK2 present within the same well as determined by ELISA.

Alternatively, the auto-phosphporylation activity of LRRK2 was assessed by the incorporation of phosphate at the S1292 residue using a phospho-specific antibody (generously provided by Genentech; see [11]). Lysate from HEK293T cells over-expressing Flag-LRRK2 was bound to Flag-coated ELISA plates in duplicate as described above, and washed extensively leaving only Flag-LRRK2 immobilized in the wells. The ELISA plates were processed in parallel for pS1292 or total LRRK2 by ELISA. The kinase reaction was performed in the absence of any added substrate, and parallel reactions performed in the absence or presence of ATP. Reactions in the absence of ATP revealed the steady-state level of S1292 auto-phosphorylation; whereas reactions performed in the presence of ATP indicated the in-well activity of LRRK2 with respect to auto-phosphorylation at this residue. The chemiluminescence signal from the pS1292 ELISA was normalized to the total LRRK2 signal in each sample to correct for variances in LRRK2 expression. As a control for the specificity of the antibody, lysate from cells expressing S1292A-LRRK2 were processed in parallel. We immunoprecipitated Flag-LRRK2 from HEK293T cells over-expressing WT, G2019S, or S1292A-LRRK2. The beads were incubated in kinase reaction buffer in the absence or presence of ATP (100 μM) for 30 min at 30°C, under constant agitation. For quantification of cellular pS1292 levels, the level of over-expressed Flag-LRRK2 was normalized for total protein levels using βeta-actin; and then the band intensity of pS1292 was expressed as a ratio to normalized Flag-LRRK2 levels.

### Assessment of LRRK2 Interaction with FADD

To determine the extent to which mutations in LRRK2 altered the interaction with the extrinsic death pathway adaptor protein FADD, we utilized two approaches. V5-tagged FADD and Flag-LRRK2 constructs were co-expressed in HEK293T cells at a ratio of 4:1 (with an excess of LRRK2) for a period of 72h. The cells were washed in cold PBS, pelleted, and resuspended in IP lysis buffer (20mM HEPES, pH 7.4; 150 mM NaCl; 0.1% NP-40; 2mM EGTA; 2 mM MgCl_2_; 10% glycerol; pH 7.2; phosphatase inhibitor cocktail and protease inhibitors [Roche]), and incubated on ice for 30 min. The cells were subjected to further disruption by a Dounce glass homogenizer and clarified by centrifugation at 13000rpm for 15 min at 4°C. In some experiments, 500 μg of clarified lysate was incubated overnight with Flag-conjugated resin (Biotool; Munich, DE), followed by washing in lysis buffer, before final elution of the protein in 2X SDS sample buffer at 95°C for 5 min. Eluate was separated by 10% SDS-PAGE, transferred to nitrocellulose, and the membranes probed with antibodies to V5 or Flag (Sigma). In parallel experiments, 15 μg of clarified lysate was bound to Flag-coated ELISA plates (as in the kinase activity experiments) for 2h at 37°C. Parallel ELISA reactions were performed to detect Flag-LRRK2 and V5-tagged FADD. The wells were washed in ELISA wash buffer (50 mM Tris, pH 7–4; 150 mM NaCl; 0.1% Tween-20), and incubated with rabbit primary antibody against LRRK2 (Abcam; clone UDD3) or V5 for 1h at room temperature, followed by HRP-conjugated anti-rabbit secondary antibody for 1h at room temperature. The wells were washed again, and incubated for 5 min with chemiluminescent substrate (Thermo Scientific). Equivalent amounts of protein lysate were separated by SDS-PAGE and probed for V5 and beta-actin, in order to normalize V5-FADD expression relative to beta-actin. The quantification is expressed as the ratio of V5-FADD chemiluminescence (normalized for total V5 expression levels) to the amount of Flag-LRRK2 captured in the ELISA plate.

### Statistical Analyses

For the statistical comparisons used, we employed a one-way ANOVA, followed by Tukey’s HSD post-hoc test for multiple comparisons. The *p* value is indicated in the Figure Legend; and the data are presented as the mean +/- standard error of the mean. The number of replications from independent experiments is provided in the Figure Legend.

## Results

### Multiple LRRK2 variants induce neuronal death

**Fig 1:**
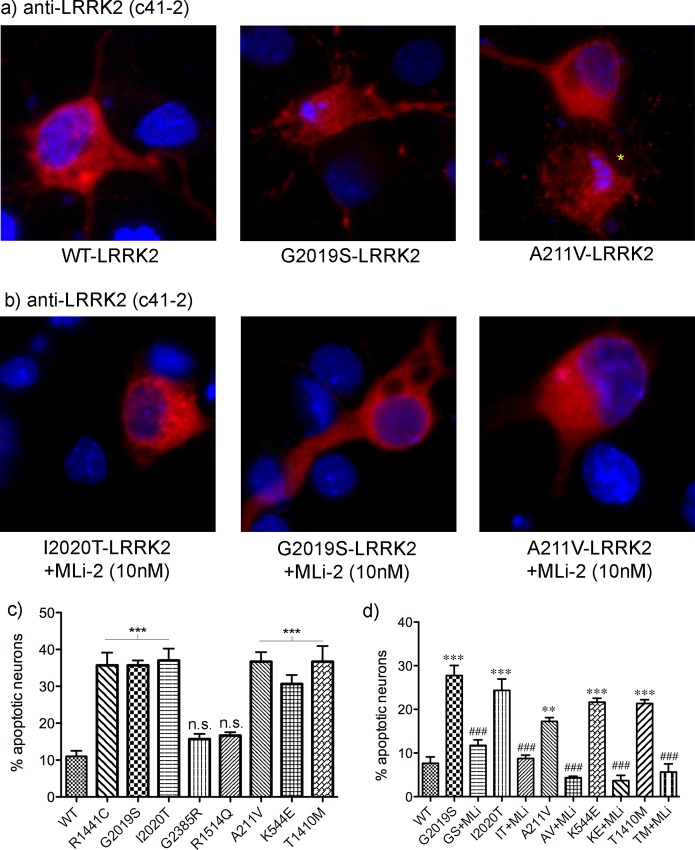
Kinase dependent apoptotic death of primary neurons expressing pathogenic or rare mutations in LRRK2. **a)** Primary rat cortical neurons were co-transfected with WT or mutant LRRK2 as indicated together with pcms-EGFP, and fixed and processed for anti-LRRK2 immunofluorescence together with Hoechst to label nuclei. Representative images are shown depicting anti-LRRK2 in red. **b)** Primary neurons as in (a) except treated with the LRRK2 kinase inhibitor MLi-2 (10nM). **c)** For quantification of apoptotic neurons, a minimum of 100 GFP-positive neurons were counted from each of three parallel coverslips per condition in a blinded fashion. Three independent transfections were conducted. Apoptotic neurons were defined as having two or more condensed nuclear fragments within a GFP-expressing neuronal profile. ** p<0.01, compared to WT; *** p<0.001, compared to WT; ### p<0.001, compared to the corresponding vehicle-treated culture.

We examined the neurotoxic potential of several variants in LRRK2 that have been identified in PD patients. Multiple cellular models have been employed to assess the induction of cell death by mutant forms of LRRK2, including cultured SH-SY5Y cells as well as primary cortical neurons [10,13,14]. We utilized primary rat cortical neuronal cultures transiently co-expressing Flag-LRRK2 and EGFP. Following 72h of expression, the neurons were fixed and the percentage of GFP-positive cells harboring classical apoptotic nuclear features (condensed, fragmented chromatin) was determined. For comparison, cells expressing pathogenic mutant LRRK2 (R1441C, G2019S, or I2020T), or non-pathogenic variants/risk factors (R1514Q or G2385R) were similarly assessed. A representative image showing the co-localization of anti-Flag immunoreactivity in GFP-positive neurons is shown in S1A Fig. Neurons expressing the pathogenic LRRK2 mutations, display nuclear features associated with classical late-stage apoptotic signaling. A representative confocal image of a control non-apoptotic neuron, and an apoptotic, caspase-3 positive neuron is shown in S1B Fig. The cellular and nuclear morphology of apoptotic neurons, as well as the accompanying caspase-3 immunoreactivity, was similar regardless of the specific LRRK2 mutation. In contrast, the R1514Q and G2385R variants did not induce apoptotic neuronal death. In (Fig 1A & 1B) representative images are shown of neurons stained with anti-LRRK2 (clone c41-2) are shown following treatment with vehicle or the highly potent LRRK2 kinase inhibitor (MLi-2, 10nM; kindly provided by Dr. Dario Alessi, The University of Dundee). We quantified the percentage of LRRK2-positive neurons with apoptotic nuclei. In neurons over-expressing WT LRRK2, we observed approximately 11% apoptotic death (Fig 1C); whereas the pathogenic LRRK2 mutations, as well as the LRRK2 variants, induced approximately 30–40% apoptotic death when assessed at 72h following expression. Treatment with the MLi-2 kinase inhibitor significantly reduced the percentage of neurons exhibiting apoptotic nuclear morphology. In addition, virtually all of the GFP/LRRK2-positive neurons that displayed classical late-stage apoptotic nuclear morphology were also labeled with antibodies for the active form of caspase-3 (see example in S1 Fig). This indicates that these less common variants identified in patients with PD are also capable of inducing the apoptotic death of cultured neurons, to a similar extent as the well-described causative mutations, and in a kinase-dependent manner.

### Differential effect of LRRK2 variants on kinase activity

**Fig 2:**
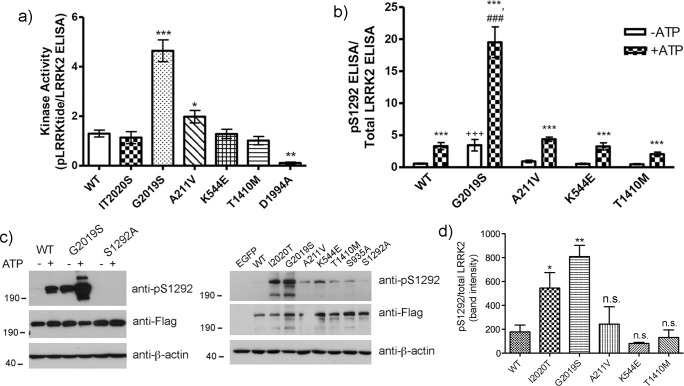
Comparison of kinase activity of LRRK2 variants and pathogenic mutants. a) Quantification of phosphorylated LRRKtide was performed by ELISA using phospho-specific LRRKtide antibodies. Subsequently, the captured Flag-LRRK2 was quantified by an ELISA using a N-terminal targeted anti-LRRK2 antibody. The amount of phosphorylation of LRRKtide was directly normalized to the amount of LRRK2 present within the same well. * p<0.05 in comparison to WT; *** p<0.001 in comparison to WT. b) To determine the in-well auto-phosphorylation activity of LRRK2 at the Ser1292 residue, kinase reactions were carried in Flag-captured ELISA plates in kinase reaction buffer in the presence or absence of 100 μM ATP, without additional substrate added. The data were normalized to parallel wells processed for total LRRK2 by ELISA. In the absence of ATP, the signal represents the basal levels of Ser1292 auto-phosphorylation; whereas in the presence of ATP, the induction of auto-phosphorylation activity at this residue is observed. The data points in (a) and (b) represent the mean of at least three independent experiments, with duplicate wells per sample within each individual experiment. c) representative Western immunoblots showing the specificity of the pS1292 antibody, the absent signal in extracts from cells expressing S1292A-LRRK2 (left blot). In the right panel, the relative degree of cellular phosphorylation levels of S1292 as determined by Western immunoblot using the same antibody, and the corresponding level of Flag-LRRK2 expression using anti-Flag antibodies. When normalized across at least 3 independent experiments, an induction in basal S1292 phosphorylation is only significantly elevated in cells expressing the pathogenic mutants I2020T or G2019S-LRRK2 (d). *** p<0.001 in comparison to the absence of ATP; ### p<0.001 in comparison to WT in presence of ATP; +++ p<0.001 in comparison to WT in absence of ATP.

The effect of the different pathological mutations on the intrinsic kinase activity of LRRK2 varies depending upon the particular mutation as well as the substrate assessed. For example, the most common causative mutation in LRRK2, G2019S, which substitutes a Ser for the conserved Gly in the DFG motif in the LRRK2 kinase domain, generally shows at least a 3-fold induction of kinase activity depending on the method of assessment (see [4] for recent review). We utilized an ELISA based assay to quantify the phosphorylation of the LRRKtide peptide substrate. In this assay, the phosphorylation of LRRKtide is normalized to the amount of LRRK2 present in the same well as determined by a novel LRRK2 ELISA. Similar to what has been reported, we find an approximate 3-fold induction of phosphorylation of LRRKtide by G2019S-LRRK2, and the absence of significant signal in cells expressing D1994A kinase dead LRRK2 (Fig 2A). In contrast, while the K544E and T1410M variants did not differ from WT in the phosphorylation of LRRKtide, the A211V induced a slight but significant enhancement of phosphorylation of this peptide (Fig 2A).

The auto-phosphorylation at Ser1292 has been employed as a proxy for LRRK2 kinase activity [11,15]. In cells expressing WT or mutant LRRK2, the kinetics of phosphorylation of the LRRKtide peptide substrate are similar to auto-phosphorylation at the Ser1292 site [15]. We assessed both basal as well as intrinsic S1292 auto-phosphorylation activity, using a phospho-specific anti-pS1292 ELISA. For the assessment of S1292 auto-phosphorylation activity, we developed a novel in-well auto-phosphorylation assay in which we compared the induction of phosphorylation at the Ser1292 site in the absence or presence of ATP. We find, similar to phosphorylation of LRRKtide, that the G2019S mutation shows an enhanced basal phosphorylation at the S1292 site, and induces an approximate 4-fold induction of phosphorylation in comparison to WT LRRK2 (Fig 2B). None of the other variants showed any significant differences compared to WT LRRK2 in this ELISA-based assay; all variants showed the expected induction of phosphorylation with ATP (Fig 2B). Immunoprecipitated Flag-LRRK2 incubated in the presence or absence of ATP shows an induction of auto-phosphorylation at this residue, which is absent in cells over-expressing S1292A-LRRK2 (Fig 2C). By Western immunoblot, we find that basal cellular levels of pS1292 are elevated in cells expressing G2019S-LRRK2 as well as I2020T-LRRK2, as has been reported [11]. For the other rare sequence variants, however, once normalized to total LRRK2 levels, we detected no significant change overall in cellular levels of pS1292 (Fig 2C and 2D). LRRK2 also auto-phosphorylates at the T1410 ROC domain site [16]. However, in our hands, using an antibody to pT1410 (Abcam; clone MJFR4-25-5) we routinely observed a positive band in cells expressing T1410M mutant LRRK2 (S2 Fig) and also occasionally in cells expressing the T1410A used as a control; thus we were not able to definitively evaluate auto-phosphorylation at this residue.

### Effect of LRRK2 sequence variants on GTP binding

**Fig 3:**
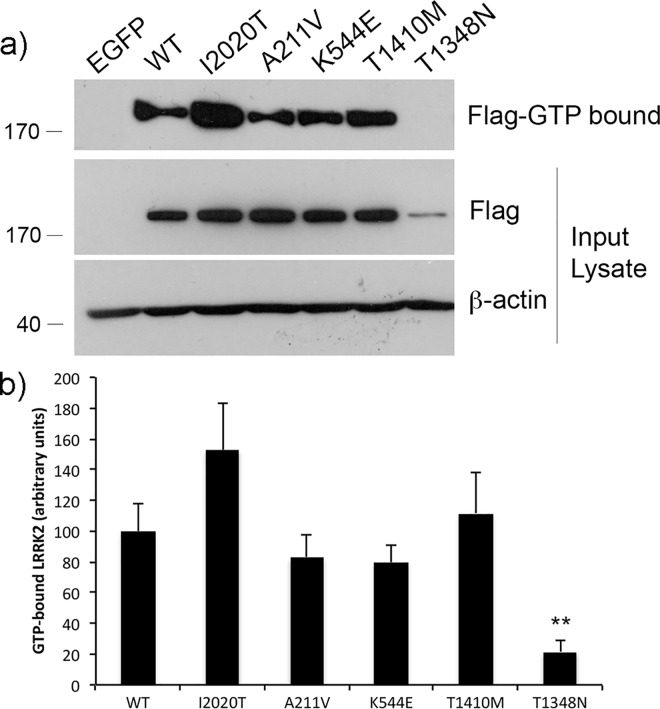
LRRK2 variants across multiple domains do not significantly alter binding to GTP. a) HEK293T cells transiently over-expressing Flag-LRRK2 (as indicated) were lysed in PBS and incubated overnight with GTP-conjugated agarose beads. The following morning, the beads were washed, eluted in sample buffer and analyzed by SDS-PAGE for the presence of GTP-bound Flag-LRRK2. The relative expression level of LRRK2 was determined in the input lysate, relative to beta-actin. b) Densitometric analyses were conducted using ImageJ from 3 independent experiments. ** p<0.01, compared to WT.

Next we determined the GTP binding capacity of the LRRK2 variants in comparison to WT or a PD-linked causative mutation. GTP binding is thought to play an important role in multiple features of LRRK2 biology, including its kinase activation as well as its propensity to dimerize. Artificial mutations in LRRK2 that inhibit its ability to bind GTP, such as T1348N, block its dimerization as well as phosphorylation of the LRRKtide substrate [17]. We find that while the I2020T pathogenic LRRK2 mutation induced a slight, but not significant, increase in the amount of GTP binding in comparison to WT, the A211V and K544E mutant forms bound GTP to a similar extent, or even moderately reduced, as WT. The ROC GTPase domain mutant, T1410M, showed a slight, but not significant increase in binding to GTP (Fig 3). As a control, the T1348N GTP-binding deficient mutant LRRK2, which is comparatively unstable in cells, shows no specific binding to GTP.

### Re-distribution of mutant LRRK2 into HMW complexes

**Fig 4:**
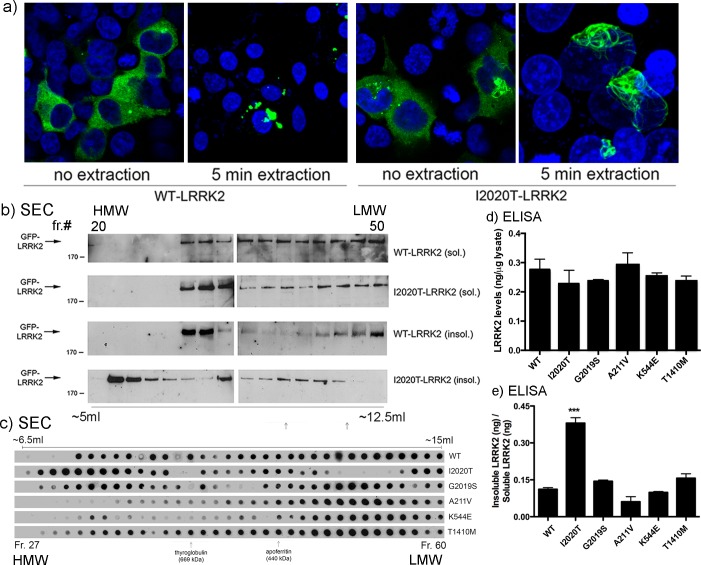
Rare LRRK2 PD-related sequence variants fail to significantly alter its oligomerization or solubility to a similar extent as pathogenic mutants. a) HEK293T cells expressing GFP-tagged I2020T-LRRK2 were extracted with 0.1% Triton X-100 for 5 min, fixed and processed for anti-GFP immunostaining with DAPI counterstaining. b) HEK293T cells were transfected and extracted as in (a), and the resulting soluble and insoluble fractions were subjected to SEC. Alternate fractions were analyzed by SDS-PAGE for the presence of GFP-tagged LRRK2. HEK293T cells transiently over-expressing Flag-LRRK2 were (c) lysed normally to produce whole-cell extracts, or (d, e) subjected to mild in situ extraction as in (a). c) Detergent-free total cell extracts of HEK293T cells subjected to SEC were analyzed by dot blot using anti-Flag antibodies to show any shift in elution towards lower or higher molecular weight complexes. Representative blots are shown (of n = 2), demonstrating in a complimentary way the differential oligomeric behavior of the different LRRK2 mutants. The approximate elution point of the native molecular weight standards thyroglobulin and apoferritin are shown as a reference only. d) The amount of LRRK2 (in ng; determined by ELISA using recombinant full-length LRRK2 as a standard) present in the total fraction, or in either the soluble or insoluble fractions was determined by ELISA. Similar levels of expression were observed for each of the mutants tested. The increased ratio of insoluble:soluble LRRK2 for the I2020T mutant (e) reflects its re-organization to extraction-resistant filamentous structures.,None of the other sequence variants significantly altered this profile in comparison to WT-LRRK2, although there was a non-significant trend for reduced solubility for the T1410M variant. ** p<0.01 compared to WT-LRRK2.

Pathogenic PD-linked mutations in LRRK2 induce a re-distribution of the protein to skein-like filamentous structures in primary cortical neurons [18], and other cell lines (e.g. [19,20]). Additionally, as previously noted, a proportion of over-expressed LRRK2 assumes a punctate aggregate-like distribution ([18] and see Fig 4A below), possibly related to the GFP epitope tag; importantly however, this distribution is unaffected by the disease-linked mutations, and thus presumed to be unrelated to the pathogenic effects of mutant LRRK2. To determine if this change in localization could be documented biochemically, we subjected lysates of HEK293T cells expressing GFP-LRRK2 to size exclusion chromatography (SEC). Pilot experiments had revealed no significant differences in the elution pattern by SEC, of WT compared to PD-mutant LRRK2 when the lysates were prepared using buffers containing 1% Triton X-100, or 1% NP-40, suggesting that these filamentous structures are sensitive to moderate to harsh detergent extraction. To help preserve any HMW complex containing LRRK2, the cells were lysed by Dounce homogenization in a detergent-free buffer (PBS). Western blot analyses revealed that the bulk of LRRK2 was detected in fractions ranging from 6.25 ml to 12.5 ml, which roughly corresponds to a MW range of 3.9 MDa to 200 kDa based on comparison to generic native molecular weight standards run under identical conditions. Each of the pathogenic disease mutants induced a shift in elution of LRRK2 towards HMW fractions (S3 Fig), with G2019S-LRRK2 showing the mildest phenotype. To more accurately assess this shift in the absence of the GFP tag, we performed an identical fractionation in cells expressing Flag-tagged LRRK2, and quantified the presence of LRRK2 within each fraction using a more sensitive ELISA. We measured the amount of LRRK2 within each fraction in reference to a standard curve established using full-length Flag-LRRK2. We find a similar shift in elution of mutant LRRK2 towards HMW fractions (S3D and S3E Fig), confirming the shift exhibited by GFP-tagged mutant LRRK2 (S3 Fig).

To further isolate the fractions that represent filamentous LRRK2, we reasoned that these structures might exhibit an altered solubility, and subjected the cells to a mild non-ionic detergent extraction. The cells were incubated in extraction buffer containing 0.1% Triton X-100 for 5 min. By immunofluorescence, cells not subjected to extraction displayed both diffuse and punctate cytoplasmic LRRK2 (Fig 4A), as well as discrete filamentous LRRK2 as previously described ([18]; Fig 4A) in cells expressing I2020T-LRRK2. Following extraction, diffuse cytoplasmic LRRK2 was lost, whereas the presence of filamentous LRRK2 was unaffected (Fig 4A). To biochemically characterize the filamentous pool of LRRK2, cells expressing WT or I2020T-LRRK2 were subjected to similar extraction and then separated by SEC. Following separation by SEC, the “insoluble” fraction of the cells remaining following extraction revealed a distinct range of HMW fractions in which I2020T-LRRK2 was present (Fig 4B, lower blot). We observed no difference in the elution of the “soluble” pool of WT or I2020T mutant LRRK2 following mild detergent extraction (Fig 4B, upper two blots). When this preparation is examined *in situ* by immunofluorescence (as in Fig 4A), the only visible pool of I2020T mutant LRRK2 that remains are the filamentous LRRK2-positive structures. It is very likely then, that the HMW fractions of I2020T-LRRK2 present following extraction are enriched in filamentous LRRK2.

In order to more quantitatively assess differences in the relative levels of “soluble” vs. “insoluble” LRRK2 following this mild extraction protocol, we turned to an ELISA-based assay. HEK293T cells expressing Flag-tagged WT, G2019S, I2020T, A211V, K544E, or T1410M-LRRK2 were subjected to mild detergent extraction as before, and each fraction bound to Flag-coated 96-well ELISA plates. As a control and to quantify the relative amounts of LRRK2 present, a standard curve comprised of human Flag-tagged WT recombinant LRRK2 was established. Total cell extracts prepared in parallel demonstrated that the total amount of over-expressed LRRK2 as measured by ELISA was equal between each group (Fig 4D). The amount of LRRK2 present within each fraction is normalized to the amount (in ng) of LRRK2 measured in total cellular extracts in reference to the standard curve of recombinant LRRK2. Expressing the relative amount of LRRK2 present in the insoluble pool proportional to the soluble pool, we find a significant quantitative induction in the amount of insoluble I2020T-LRRK2, as would be expected from a re-distribution into cytoplasmic filaments (Fig 4E), and consistent with the elution profile from the SEC column. In contrast, the other mutant forms of LRRK2 did not show a significantly different alteration in solubility compared to WT-LRRK2 (Fig 4E). There was a slight, but significant, trend towards reduced solubility in the G2019S form of LRRK2; whereas A211V displayed slightly greater solubility, and K544E was indistinguishable from WT. The small increase in insolubility observed for T1410M LRRK2 did not reach the level of statistical significance (p = 0.056). This pattern was also apparent qualitatively in SEC-fractions assessed by dot-blot membranes probed for anti-Flag (a representative dot blot is shown in Fig 4C). Overall, our results point to a divergence between the clear pathogenic mutations and the variants examined here, in terms of the re-distribution of cellular LRRK2. In fact, even among the pathogenic mutant forms, there is no clear pattern of alterations in solubility or localization; with the I2020T mutation inducing a significant increase in detergent-resistant filamentous structures and robust shift in elution towards HMW fractions, while mutation at the adjacent residue (G2019S) results in a milder phenotype. While the R1441C ([18] and I2020T (Fig 4 and [18]) forms of LRRK2, to varying degrees, re-distribute within different cytoplasmic compartments into skein-like filaments, the two N-terminal LRRK2 variants, A211V and K544E, do not possess this feature. The G2019S mutant and the T1410M variant occupy an intermediate position and likely promote redistribution to a smaller degree.

### Interaction of LRRK2 with the death adaptor protein FADD

**Fig 5:**
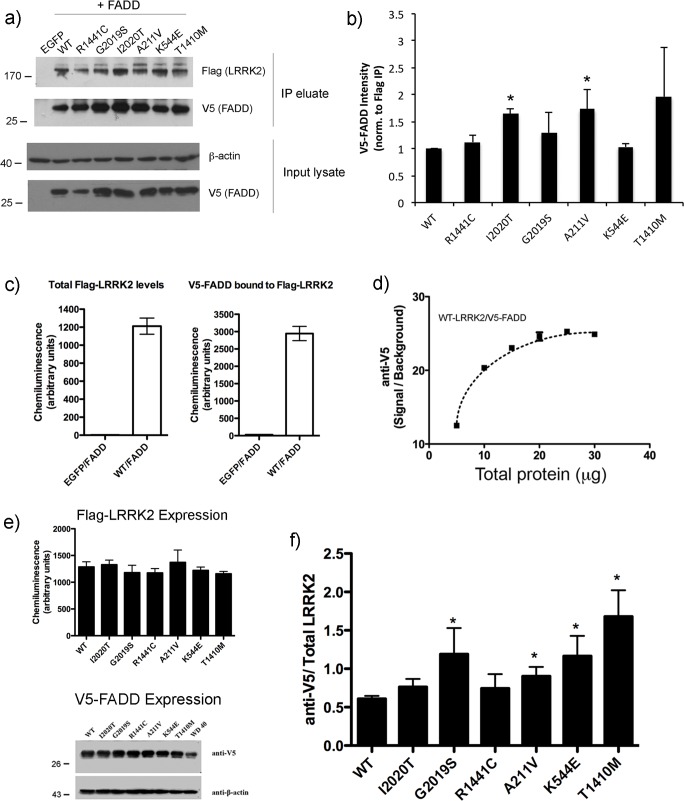
Interaction with the death adaptor protein FADD is a common feature of LRRK2 sequence variants that elicit neurotoxicity. HEK293T cells were transiently transfected with Flag-LRRK2 and V5-FADD at a ratio of 4:1, and after 3 days of expression clarified cell extracts were incubated overnight with M2-Flag resin (Sigma), followed by washing and elution in 2X SDS buffer. The eluates and input lysates were separated by SDS-PAGE and the membranes probed with anti-V5 (rabbit) or anti-Flag antibodies (rabbit). (a) A representative image of the Western immunoblot is shown to demonstrate the interaction between LRRK2 and FADD. (b) Quantification of band intensity from three independent experiments. The value of the V5 immunoreactive band was normalized to the amount of Flag-LRRK2 immunoprecipitated in each sample. * p<0.05 compared to WT-LRRK2. To more precisely quantify the change in interaction between LRRK2 and FADD, we developed an ELISA-based approach. c) Lysates from cells expressing V5-FADD and either EGFP or Flag-WT-LRRK2 were incubated in Flag-coated ELISA plates. In the left plot, LRRK2 (clone UDD3) was detected only in lysates transfected with Flag-LRRK2. To confirm that V5-FADD does not bind non-specifically to the ELISA plate independently of the presence of LRRK2, we processed parallel ELISA wells using V5 as the detection antibody (right plot), confirming that V5-FADD can only be detected with co-expressed with LRRK2. d) To determine the linearity of the signal, we incubated increasing amounts of lysate from cells co-expressing Flag-tagged WT-LRRK2. Using V5 as the detection antibody to detect the presence of FADD, we observe an increase in chemiluminescence signal between 5 and 20 μg of cell extract. In experiments quantifying the interaction of FADD with mutant LRRK2, we assessed 15 μg of cell extract loaded in each well. e) The presence of LRRK2 was determined by sandwich ELISA using anti-LRRK2 (clone UDD3) as the detection antibody. Lysates from cells co-expressing LRRK2 and V5-FADD were analyzed by SDS-PAGE to confirm FADD expression in all samples. f) While each of the three pathogenic mutant forms of LRRK2 showed slightly increased interaction with FADD using this approach, only the G2019S-LRRK2 was significantly greater than WT LRRK2. Similarly, each of the three rare LRRK2 sequence variants showed significantly greater interaction with FADD. * p<0.05 compared to WT-LRRK2.

Some pathogenic mutations in LRRK2 enhance the baseline interaction with the extrinsic death adaptor protein FADD [10]. This leads to the recruitment and activation of caspase-8, which is indispensible for the death of primary neurons expressing mutant LRRK2 [10]. We asked if there was a similar enhancement of the interaction with FADD in cells expressing A211V, K544E, or T1410M-LRRK2. Cells co-expressing Flag-tagged LRRK2 (as indicated) and V5-tagged FADD, were subjected to immunoprecipitation with anti-Flag-coated resin, and the co-precipitation of FADD was determined by Western immunoblot for V5. A representative immunoblot showing co-precipitation of V5-FADD with Flag-LRRK2 is shown in Fig 5(A), showing a slight but variable increase in the interaction between FADD and many of the mutants tested. We quantified the increase in the amount of V5-FADD co-eluting with immunoprecipitated Flag-LRRK2; however, due to the large variability introduced in the measurements of band intensities from triplicate experiments, only the I2020T-LRRK2 mutant and A211V-LRRK2 variant reached a statistical significance (Fig 5B).

For more precise quantification, we employed an ELISA-based approach in which Flag-tagged LRRK2 complexes are captured in a 96-well plate and the presence of V5-FADD detected by an anti-V5 ELISA. For comparison, three pathogenic mutant forms of LRRK2 (R1441C, G2019S, and I2020T) were included. We performed a number of experiments to confirm the specificity of the Flag-LRRK2/V5-FADD ELISA approach. First, we incubated lysate from cells co-expressing EGFP and V5-FADD or WT-LRRK and V5-FADD on plates coated with anti-Flag antibodies. We performed a sandwich ELISA using an antibody to detect total LRRK2 present in the well (rabbit monoclonal, clone UDD3). Specific signal was detected only from extract prepared from cells expressing Flag-LRRK2 (Fig 5C, left plot). With the same cell extracts, we performed an ELISA using anti-V5 as the detection antibody, and measured specific V5-FADD present only in extracts from cells co-transfected with LRRK2 and FADD (Fig 5C, right plot). Next, we incubated increasing amounts of lysate from cells co-expressing Flag-tagged WT-LRRK2 and V5-FADD in wells coated with anti-Flag, and performed the V5 ELISA as described to determine the linear range of specific detection of FADD. In our experiments to quantify the amount of FADD co-purifying with LRRK2, we loaded 15 μg of total cell lysate, within the linear portion of the curve (Fig 5D). For each of the mutants tested, we verified equal LRRK2 and FADD expression by Western immunoblotting, and quantitatively by ELISA (Fig 5E).

We find, using this approach, that G2019S-LRRK2 significantly enhances the interaction with FADD in comparison to WT-LRRK2 (Fig 5F). The other kinase domain mutant, I2020T, and the ROC-GTPase domain mutant R1441C, exhibited a trend, which did not reach statistical significance, towards increased interaction with FADD compared to WT. Interestingly, each of the rare LRRK2 variants tested also showed an enhanced interaction with FADD compared to WT LRRK2 (Fig 5F), suggesting that the interaction of LRRK2 with FADD, either by enhancing binding or through another mechanism, is an important step in the induction of neuronal death.

**Fig 6:**
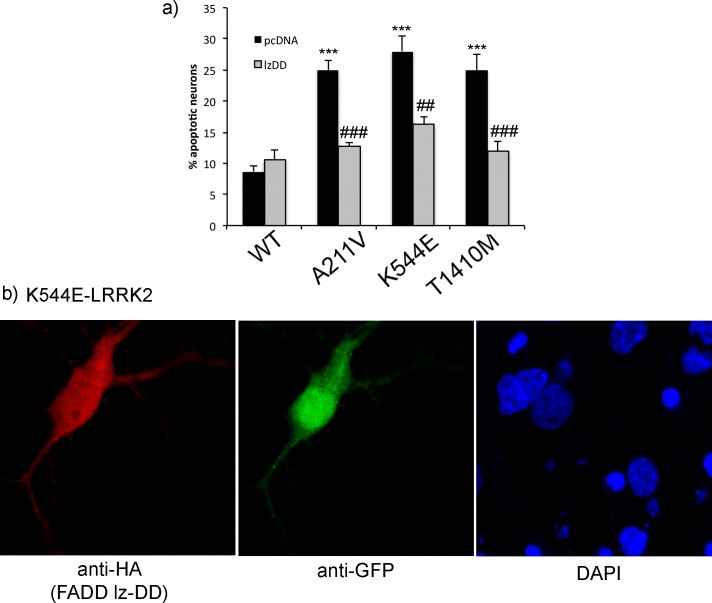
Disrupting FADD-dependent signaling in primary neurons is neuroprotective. a) Primary cortical neurons were co-transfected with an EGFP-pCMS reporter vector, and the LRRK2 mutant together with the FADD dominant negative (HA-tagged leucine-zipper FADD death domain; lzDD) or empty pcDNA vector as a control. The percentage of GFP-positive neurons containing apoptotic nuclear features (defined as above) was determined 72h following transfection. The presence of the dimeric FADD death domain significantly reduced neuronal apoptotic death in cultures expressing each of the LRRK2 variants. b) A representative image of primary neurons expressing Flag-K544E-LRRK2 with the lz-DD fragment, co-stained for ant-GFP and anti-HA (red). *** p<0.001 compared to WT LRRK2; ## p<0.01 compared to pcDNA; ### p<0.001 compared to pcDNA.

The interaction of LRRK2 with FADD was previously mapped to the death domain of FADD, and is particularly stronger when this domain is in a dimeric state [10]. In fact, overexpression of a dominant negative form of FADD, comprised of a leucine-zipper enforced dimer of the isolated death domain (lz-DD), is neuroprotective in a cellular model of mutant LRRK2-induced neuronal loss where cortical neurons co-expressing R1441C-, G2019S, or I2020T-LRRK2 together with this dominant negative fragment exhibit greater survival [10]. To determine if the enhanced interaction of these rare LRRK2 variants with FADD was functionally significant for the induction of neuronal death, we over-expressed the FADD dominant negative and assessed the proportion of LRRK2-expressing neurons with apoptotic features. As was previously reported for neuronal death induced by the pathogenic LRRK2 mutants [10], we find here that the lz-DD dominant negative FADD significantly prevents the induction of apoptotic death in primary cortical neurons expressing mutant LRRK2 (Fig 6A). Finally, to investigate whether the interaction with FADD was modulated by LRRK2 kinase activity, HEK293T cells co-expressing WT or mutant LRRK2 together with FADD were treated with the MLi-2 LRRK2 kinase inhibitor. However, as has recently been reported [21], pharmacological inhibition of LRRK2 kinase activity with inhibitors such as MLi-2 markedly de-stabilize the protein, due to rapid de-phosphorylation, making reliable determinations of the interaction with FADD difficult. Although not quantitative, the immunofluorescent anti-LRRK2 (c41-2) signal in primary neurons transiently over-expressing LRRK2 did not appear to be significantly lowered by MLi-2 treatment during the course of the experiment (see Fig 1); however in these experiments the immunofluorescence labeling was optimized for the purposes of counting and imaging, and not quantification of expression levels.

## Discussion

Despite the large number of sequence variants identified in patients with PD, only a few are causative for the disease; and even still the penetrance is not complete [15,22]. In the Greek population in particular, mutations in LRRK2 in PD patients are rare [23–26]. In 2006, Spanaki and colleagues described a family bearing a His substitution at the Arg1441 residue, in which one affected member presented an unusual progression from typical PD to progressive supranuclear palsy [27]. Since this mutation existed at a residue associated with well-described pathogenic mutations, we elected not to include this in our study. Conversely, for those novel variants that have been described, the genetic evidence for pathogenicity is unclear. For example, concerning the A211V and K544E variants that have been identified in a single family each; there is a lack of segregation for the A211V, and the K544E family has an affected member that does not carry this mutation in LRRK2 [26]. In contrast, in a larger genetic study, Ross and colleagues characterized the A211V as potentially protective [8]. It is for this reason that we undertook this study to examine in a cellular model, several phenotypes that have been described thus far in studies of PD-linked mutant LRRK2. Complicating these analyses is the fact that major phenotypic differences exist even between the known pathogenic mutant forms of LRRK2, however the common element to each of these mutants is the induction of neuronal death in primary cortical neurons. We report a similar induction of neuronal death by three rare variants of LRRK2 found in Greek PD patients, but not healthy controls, nor in other variants excluded as being pathogenic. Multiple groups have observed the death of primary cultured neurons, cultured neuronal-like cells, and *in vivo* in dopaminergic neurons of the ventral midbrain induced by over-expression of pathogenic mutant forms of LRRK2 (e.g. [18,28,29]). The mode of death appears to be an apoptotic one, requiring the activation of caspases.

Neuronal death induced by most, if not all, mutant forms of LRRK2 is also dependent upon kinase activity, as kinase-inactivating mutations as well as pharmacological inhibitors of LRRK2 kinase activity are protective (e.g [18]). Similarly, in the present work we find that pharmacological inhibition of LRRK2 kinase activity blocks neuronal death induced by the other rare sequence variants studied, in addition to the known pathogenic LRRK2 mutations. Whether this is strictly due to a loss of phosphorylation by LRRK2 of one (or more) substrates remains to be definitively established, as inhibition of LRRK2 kinase activity, either genetically or pharmacologically, can also alter both the stability and localization of the protein [13,30], and perhaps thus its ability to induce neuronal death. This phenomenon appears to be linked to de-phosphorylation of LRRK2 itself, which is accompanied by increased K63 and K48 ubiquitination and degradation [21,31]. In should be noted however, that in primary neurons treated with MLi-2, we did not observe, at a qualitative level by immunofluorescence, a dramatic reduction in the levels of over-expressed WT or mutant LRRK2.

We developed a sensitive ELISA-based assay that simultaneously measures the phosphorylation of peptide substrates (e.g. LRRKtide) as well as the relative amounts of LRRK2 present. We find, as others have shown, that the G2019S mutation induces a robust phosphorylation of this peptide substrate in comparison to WT LRRK2. Alternatively, while some groups have reported an increase in activity (e.g. [32,33]), we find no significant difference in phosphorylation of LRRKtide by the I2020T form of LRRK2 compared to WT, despite auto-phosphorylation at the Ser1292 residue being significantly elevated. Similarly for the K544E and T1410M LRRK2 variants; there is no alteration in the phosphorylation of the LRRKtide substrate. However, the A211V variant does induce a slight, yet significant, induction in phosphorylation of this substrate, although not to the extent of the G2019S form.

In the absence of confirmed *in vivo* protein substrates of LRRK2 kinase activity, apart from the recently identified Rab GTPase phospho-substrates [9], the phosphorylation of LRRK2 itself is frequently used as a representation of its kinase function. This phosphorylation can occur via direct auto-phosphorylation at multiple sites throughout the protein, or at a cluster of Ser residues located in the N-terminal domain of LRRK2. Phosphorylation at these residues (including Ser910, Ser935, Ser955, and Ser973) does not occur via direct auto-phosphorylation by LRRK2; however it has been shown in cellular systems that inhibition of LRRK2 kinase activity with pharmacological inhibitors induce de-phosphorylation at these sites, implicating other kinases in this particular modification (e.g. [34–37]). Phosphorylation at Ser910 and Ser935 is not significantly affected by the A211V, K544E, or T1410M variants, similar to G2019S [36]. The R1441G and I2020T mutations in contrast displayed markedly reduced phosphorylation at these sites [36]. Using our novel ELISA-based auto-phosphorylation assay, we find only the G2019S mutation significantly enhances auto-phosphorylation activity at the Ser1292 residue; whereas none of the other sequence variants significantly altered activity at this site. In this assay, we are measuring the *in vitro* activity of the purified enzyme, rather than its auto-phosphorylation status at the time of cell lysis.

Certain pathogenic mutations have been shown by multiple groups to cause the re-distribution of LRRK2 to cytoplasmic skein-like filaments (e.g. [18–20]). These are reminiscent of the so-called Death Effector Filaments (DEF’s) initially described by Siegel and colleagues [38] in TNF-receptor superfamily and extrinsic death pathway components. This redistribution can also occur following loss of 14-3-3 binding upon treatment with pharmacological kinase inhibitors (e.g. [35,36,39,40]). Using SEC combined with a mild extraction of soluble cytoplasmic proteins, we have isolated fractions of mutant LRRK2 corresponding to these oligomeric filamentous species. While this approach alone cannot discriminate between LRRK2 oligomers and mutant LRRK2 bound to other proteins, when taken in the context of previous work showing a) the increased co-immunoprecpitation of mutant LRRK2 [18]); and b) the increased formation of filamentous structures (present work, [18,20]), we believe that strong evidence now exists pointing to enhanced oligomerization of mutant LRRK2. A recent report by Ito and Iwatsubo [41] suggests that the native “p600” species of LRRK2 commonly identified as a LRRK2 dimer (e.g. [42,43]) might in fact represent LRRK2 monomer. In addition, upon forced dimerization of two Fv-LRRK2 fusion proteins with the AP20187 ligand, Ito and colleagues showed that LRRK2 localization was not altered and kinase activity was unchanged [41]; however this should be taken with caution as the ligand treatment may have induced a non-physiologic dimerization of LRRK2. In the present study we find a stark contrast in the solubility and SEC-elution profile of these two variants in comparison to the pathogenic I2020T mutant form of LRRK2 (see Fig 6), which coupled with the findings of Nichols and colleagues [36] is consistent with the notion that the strength of the interaction between LRRK2 and 14-3-3 is inversely related to its oligomerization.

The activation of components of the extrinsic death pathway is a necessary step for the apoptotic death of cultured primary neurons expressing pathogenic mutant LRRK2 [10]. While WT LRRK2 at baseline exhibits a strong interaction with the death-domain adaptor protein FADD, the mechanism by which mutant LRRK2 “activates” FADD leading to the recruitment and activation of caspase-8 remains unclear. Since the induction of neuronal death in primary cortical neurons was observed at a similar rate following expression of the three selected LRRK2 variants compare to known pathogenic mutants, we then asked whether this was accompanied by an altered association with FADD. Indeed, each of the variants showed an enhanced interaction with FADD, as does G2019S-LRRK2; whereas the I2020T and R1441C mutants showed only a non-significant trend towards increased interaction. Furthermore, over-expression of a dominant negative FADD, consisting of a dimeric death domain and lacking the death-effector domain which normally recruits caspase-8 in classical extrinsic death signaling, blocked apoptotic death induced by these rare LRRK2 variants, similar to that previously reported for pathogenic mutant LRRK2 [10]. It had previously been shown that genetic ablation of LRRK2 kinase activity (with the K1906R mutation) reduced the interaction of pathogenic mutant LRRK2 to baseline levels similar to WT [10]. We examined the interaction of FADD with LRRK2 in the presence of the MLi-2 kinase inhibitor used in primary neurons. We found that levels of over-expressed Flag-LRRK2 in HEK293T cells were significantly reduced (data not shown) in the presence of MLi-2, as has recently been reported [21]. Since the sequence variants share a similar degree of sensitivity to protection against neuronal death by the dimeric FADD death domain (lz-DD) as the pathogenic mutants, we predict that double mutants containing a kinase-inactivating mutation (e.g. K1906R) would also normalize the interaction with FADD. Thus, while an increase in the physical association between mutant LRRK2 and FADD does not appear to be the critical factor in the activation of this death pathway, signaling through the FADD-dependent extrinsic pathway is a shared requirement for mutations in LRRK2 that are neurotoxic.

That the three sequence variants examined here do not fully recapitulate all the phenotypic properties of the known pathogenic mutations in LRRK2 does not necessarily exclude their classification as such; nor do we intend to imply that the properties investigated in the current study represent the only salient factors in determining pathogenicity in a cellular model. Indeed, even within the different mutations clearly segregating with the disease, significant phenotypic differences are observed. Notably, the effects of the different mutations on the enzymatic activities of LRRK2 diverge markedly. While neurotoxicity induced by mutant LRRK2 is dependent upon both GTPase and kinase activities [14,18,44,45], the different mutations affect these processes in distinct ways. The kinase domain mutant G2019S induces a robust increase in phosphorylation of multiple substrates of LRRK2, likely due to its re-arrangement of the conserved DYG motif in the activation loop into an active “DYG-in” state; whereas mutation of the adjacent Ile results in a more moderate change in activity, depending upon the substrate examined and the methodology used (see [46] for review). Likewise, kinase activity assessments of the R1441C/G and Y1699C mutants failed to demonstrate consistent alterations in activity [46]. Additionally, the G2385R variant that is associated with an elevated risk of developing PD, not only shows reduced kinase activity compared to WT LRRK2, but can also reverse the induction of autophosphorylation or phosphorylation of LRRKtide seen with the G2019S pathogenic mutation [40]. The recent identification of members of the Rab GTPase family of proteins as kinase substrates of LRRK2 [9] shed new light on the complex nature of the assessment of LRRK2 kinase activity. Here, most disease-linked mutations in LRRK2 showed a robust increase in phosphorylation of several Rab proteins, to a greater extent than even G2019S [9]. As this family of GTPases are critically involved in the regulation of virtually all intracellular vesicular trafficking, including the formation and maturation of lysosomal vesicles, their involvement in apoptotic signaling in the context of phosphorylation by mutant LRRK2 may also be critical especially in the late stages or neuronal death and/or removal of dysfunctional mitochondria. Further, the interaction between LRRK2 and 14-3-3 proteins is crucial to its function and cellular localization [35,36]; however, whether 14-3-3 binding to LRRK2 suppresses or promotes death signaling remains to be determined. Over-expression studies have reported that certain pathogenic mutant forms of LRRK2 show reduced binding to 14-3-3 [36], suggesting a potential suppressive role for 14-3-3; however whether there are distinct pools of LRRK2 within the cell/neurons that retain binding and trigger cell death pathways are not known. The extent of neuronal apoptotic death induced by PD-linked mutant forms of LRRK2 is roughly equivalent, and equally dependent upon intact kinase activity [14,18,44]. This highlights the incomplete understanding of LRRK2 kinase function. Thus, while the precise signaling mechanisms triggered by the different mutants and variants in LRRK2 may differ, the net result appears to be the convergence on a common pathway involving elements of the extrinsic death cascade.

## Supporting Information

S1 Fig Colocalization between co-transfected EGFP and Flag-tagged LRRK2 in primary neurons. **a)** Primary embryonic cortical neurons were c-transfected with Flag-LRRK2 (WT or mutant) and pcms-EGFP at a ratio of 4:1. 72h following transfection, the cells were fixed and immunostained for anti-GFP and anti-Flag, together with DAPI to label nuclei. A representative image is shown of neurons expressing either WT or G2019S-LRRK2. The GFP/Flag-positive neurons expressing G2019S-LRRK2 exhibits an apoptotic nuclear morphology. We estimated the percentage of GFP-positive neurons to be labeled with anti-flag antibodies to be in excess of 85%. **b)** Primary rat embryonic cortical neurons were co-transfected with the indicated Flag-tagged LRRK2 construct and pcms-EGFP. Seventy-two hr following expression, the neurons were fixed and processed for double immunofluorescence with GFP and active caspase-3 antibodies, together with Hoechst to label nuclei. Representative images of control (WT-LRRK2) or late-stage degenerating neurons expressing mutant LRRK2 are shown depicting GFP (left, in green), active caspase-3 (middle, in red), and the appearance of apoptotic nuclei in neurons (Hoechst, right panel).
(TIF)

S2 Fig a) HEK293T cells expressing Flag-LRRK2 as indicated were lysed and subjected to SDS-PAGE and probed for phospho-T1410, phospho-S935, or Flag, for total LRRK2. Some cells were treated with 3μM LRRK2-IN1 overnight prior to lysis. b) Chromatogram confirming the C to T base-change resulting in the Met substitution for Thr at position 1410 of human LRRK2.
(TIF)

S3 Fig Oligomeric mutant LRRK2 elutes in HMW fractions. a) Lysate from HEK293T cells expressing WT or mutant LRRK2 was separated by SEC using a Superose 6 10/300 column. Fractions ranging approximately 200kDa to 3.9 MDa were analyzed by SDS-PAGE for the presence of GFP-tagged LRRK2. b) For quantification of the LRRK2 present in each fraction, triplicate blots were scanned and the band intensity measured by ImageJ, and binned into groups of 5 adjacent fractions as indicated. * p<0.05, compared to WT. We performed similar fractionations using Flag-tagged LRRK2. c) We estimated with amount of Flag-LRRK2 in ng/μl for each fraction based on extrapolation against a standard curve established with full-length recombinant Flag-LRRK2. d) HEK293T cells were transiently transfected with Flag-tagged WT or mutant LRRK2, and the amount of LRRK2 present in each fraction was determined by ELISA. In (e) each of the individual pathogenic mutants are plotted against WT-LRRK2 or cells expressing EGFP as a control. Each of the pathogenic mutants induced a shift in elution of LRRK2 towards HMW factions, suggestive of increased oligomerization.
(TIF)
