# Supplementary figures and images for: Activation of FADD-Dependent Neuronal Death Pathways as a Predictor of Pathogenicity for LRRK2 Mutations
Source: PLoS One. 2016 Nov 10;11(11):e0166053. doi: 10.1371/journal.pone.0166053 (PMC5104429; doi:10.1371/journal.pone.0166053)

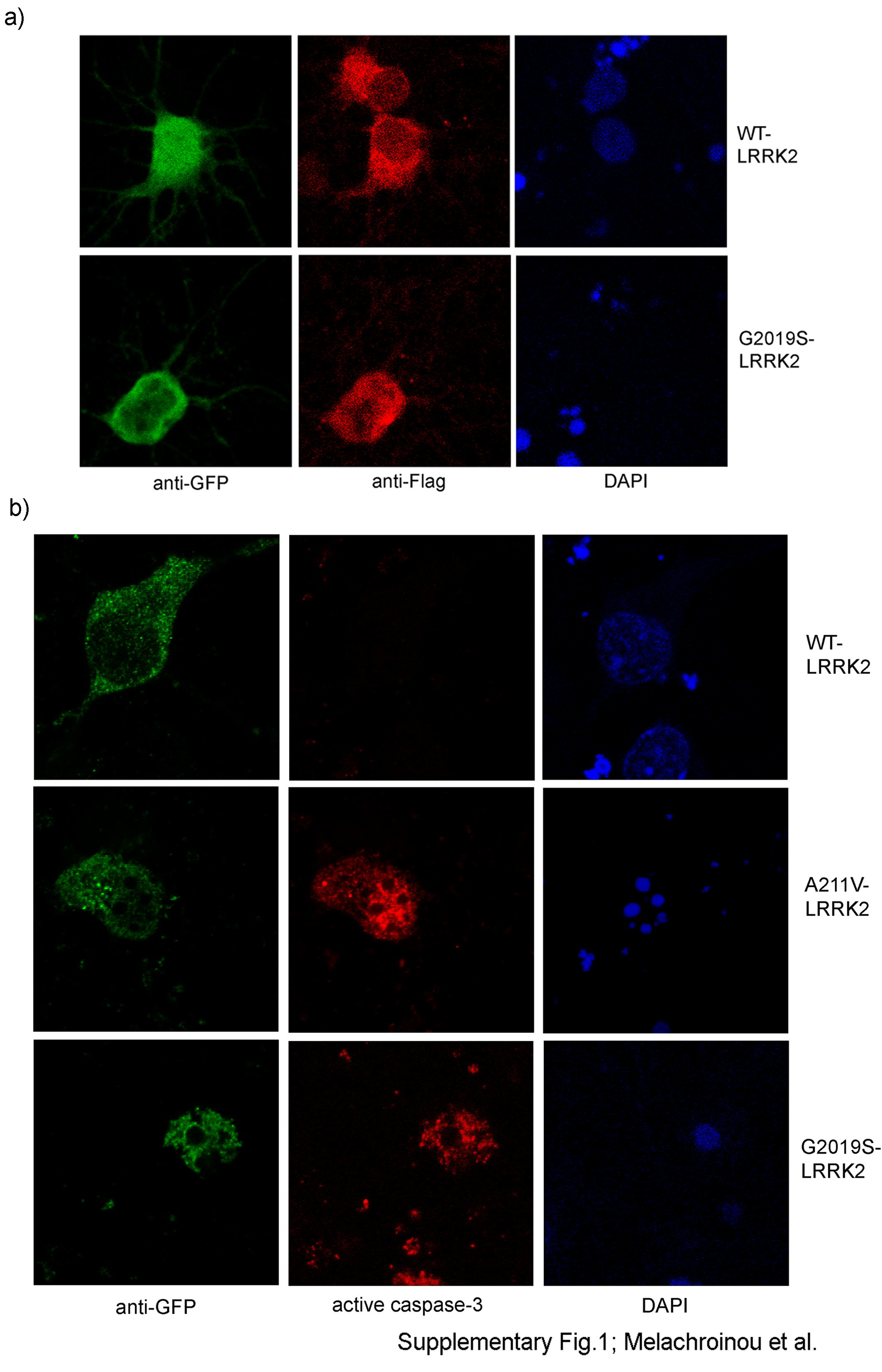

Supplement: S1 Fig — a) Primary embryonic cortical neurons were c-transfected with Flag-LRRK2 (WT or mutant) and pcms-EGFP at a ratio of 4:1. 72h following transfection, the cells were fixed and immunostained for anti-GFP and anti-Flag, together with DAPI to label nuclei. A representative image is shown of neurons expressing either WT or G2019S-LRRK2. The GFP/Flag-positive neurons expressing G2019S-LRRK2 exhibits an apoptotic nuclear morphology. We estimated the percentage of GFP-positive neurons to be labeled with anti-flag antibodies to be in excess of 85%. b) Primary rat embryonic cortical neurons were co-transfected with the indicated Flag-tagged LRRK2 construct and pcms-EGFP. Seventy-two hr following expression, the neurons were fixed and processed for double immunofluorescence with GFP and active caspase-3 antibodies, together with Hoechst to label nuclei. Representative images of control (WT-LRRK2) or late-stage degenerating neurons expressing mutant LRRK2 are shown depicting GFP (left, in green), active caspase-3 (middle, in red), and the appearance of apoptotic nuclei in neurons (Hoechst, right panel). (TIF) [file pone.0166053.s001.tif]

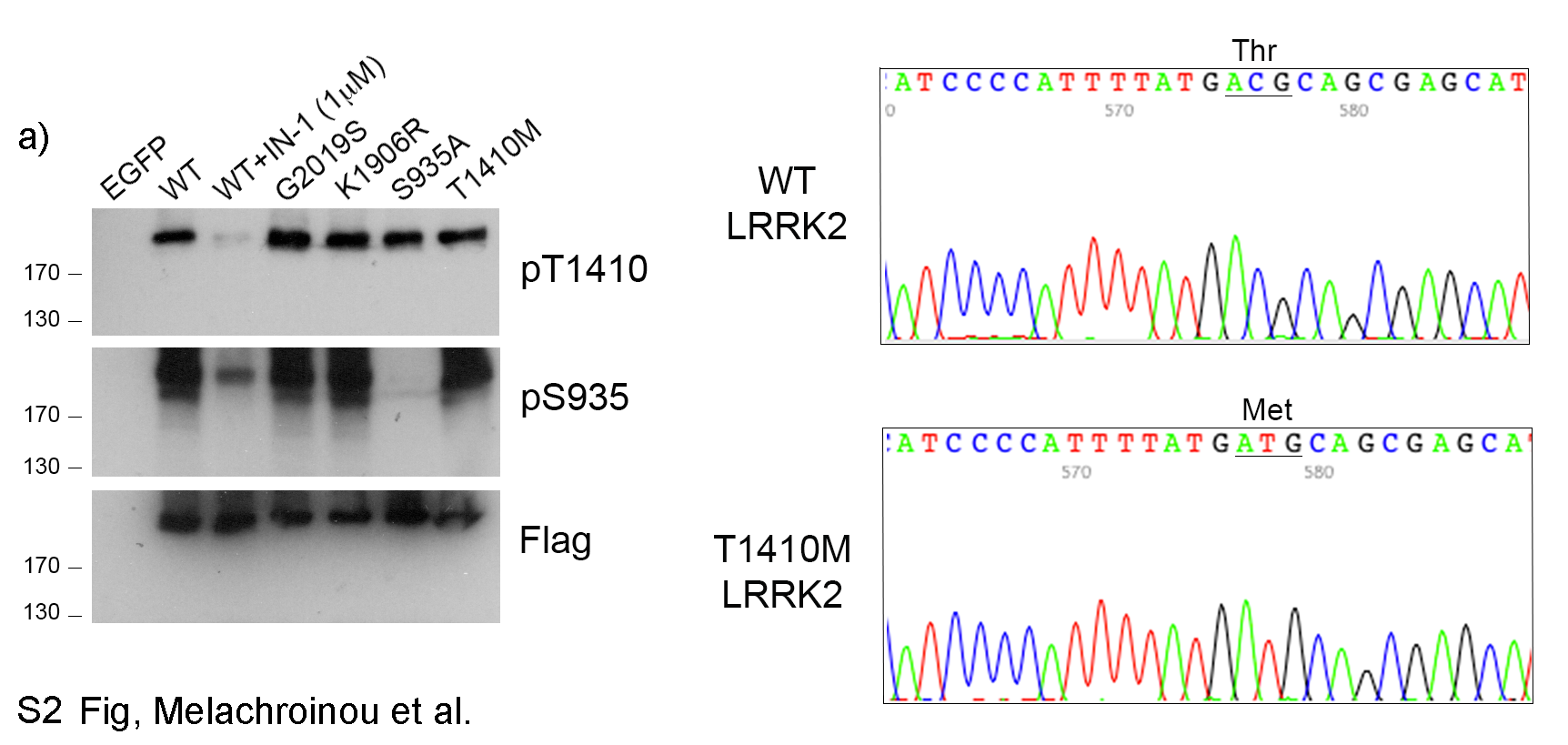

Supplement: S2 Fig — a) HEK293T cells expressing Flag-LRRK2 as indicated were lysed and subjected to SDS-PAGE and probed for phospho-T1410, phospho-S935, or Flag, for total LRRK2. Some cells were treated with 3μM LRRK2-IN1 overnight prior to lysis. b) Chromatogram confirming the C to T base-change resulting in the Met substitution for Thr at position 1410 of human LRRK2. (TIF) [file pone.0166053.s002.tif]

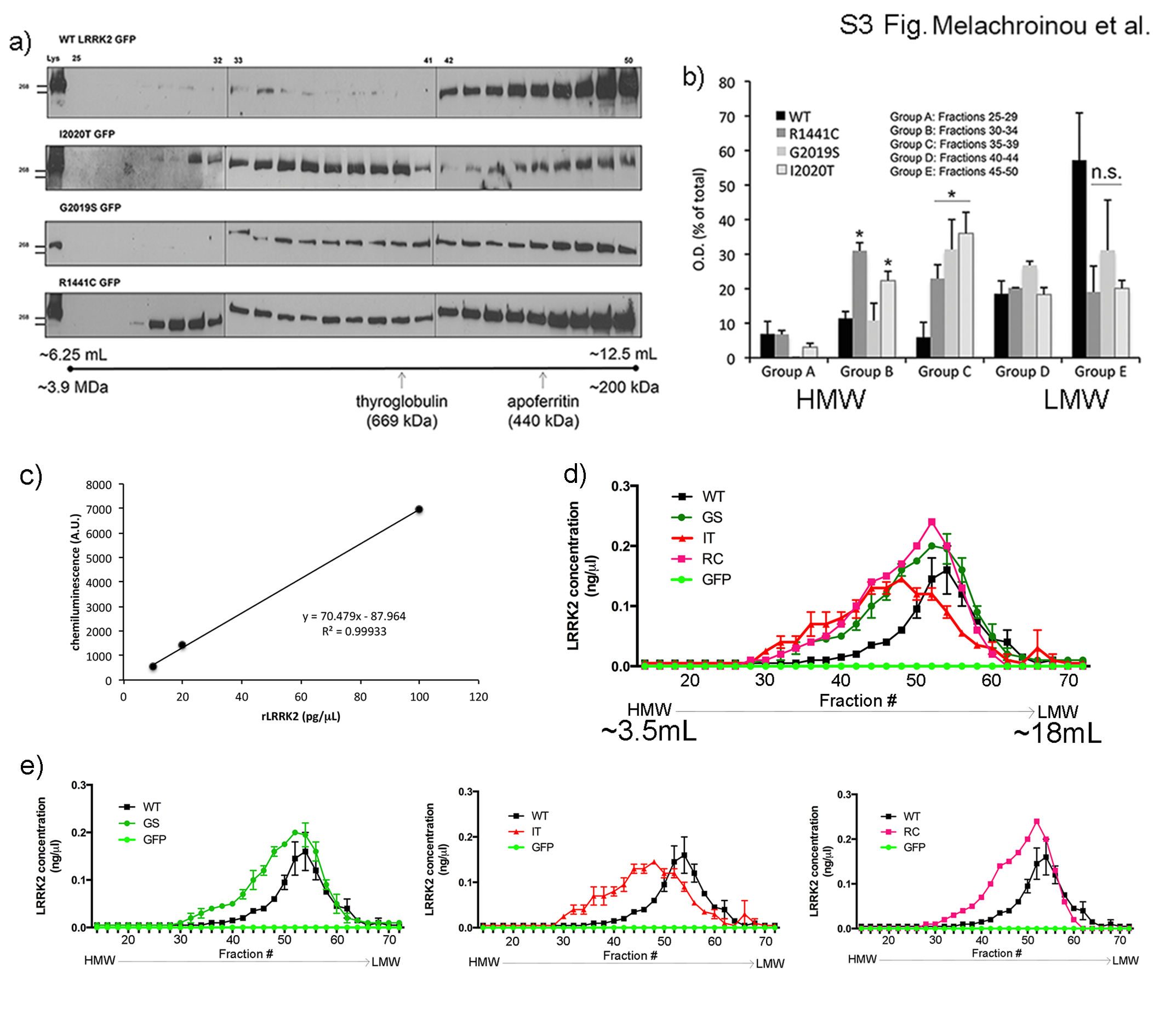

Supplement: S3 Fig — a) Lysate from HEK293T cells expressing WT or mutant LRRK2 was separated by SEC using a Superose 6 10/300 column. Fractions ranging approximately 200kDa to 3.9 MDa were analyzed by SDS-PAGE for the presence of GFP-tagged LRRK2. b) For quantification of the LRRK2 present in each fraction, triplicate blots were scanned and the band intensity measured by ImageJ, and binned into groups of 5 adjacent fractions as indicated. * p<0.05, compared to WT. We performed similar fractionations using Flag-tagged LRRK2. c) We estimated with amount of Flag-LRRK2 in ng/μl for each fraction based on extrapolation against a standard curve established with full-length recombinant Flag-LRRK2. d) HEK293T cells were transiently transfected with Flag-tagged WT or mutant LRRK2, and the amount of LRRK2 present in each fraction was determined by ELISA. In (e) each of the individual pathogenic mutants are plotted against WT-LRRK2 or cells expressing EGFP as a control. Each of the pathogenic mutants induced a shift in elution of LRRK2 towards HMW factions, suggestive of increased oligomerization. (TIF) [file pone.0166053.s003.tif]
